# Analysis of changes in microRNA expression profiles in response to the troxerutin-mediated antioxidant effect in human dermal papilla cells

**DOI:** 10.3892/mmr.2015.3717

**Published:** 2015-05-04

**Authors:** KYUNG MI LIM, SUNGKWAN AN, OK-KYU LEE, MYUNG JOO LEE, JEONG PYO LEE, KWANG SIK LEE, GHANG TAI LEE, KUN KOOK LEE, SEUNGHEE BAE

**Affiliations:** 1 Molecular-Targeted Drug Research Center and Korea Institute for Skin and Clinical Sciences, Konkuk University, Seoul 143-701, Republic of Korea; 2 Coreana Cosmetics Co., Ltd., Cheonan, Chungcheong 330-882, Republic of Korea

**Keywords:** microRNA, troxerutin, antioxidant effect, dermal papilla cells, senescence

## Abstract

Dermal papilla (DP) cells function as important regulators of the hair growth cycle. The loss of these cells is a primary cause of diseases characterized by hair loss, including alopecia, and evidence has revealed significantly increased levels of reactive oxygen species (ROS) in hair tissue and DP cells in the balding population. In the present study, troxerutin, a flavonoid derivative of rutin, was demonstrated to have a protective effect against H_2_O_2_-mediated cellular damage in human DP (HDP) cells. Biochemical assays revealed that pretreatment with troxerutin exerted a protective effect against H_2_O_2_-induced loss of cell viability and H_2_O_2_ induced cell death. Further experiments confirmed that troxerutin inhibited the H_2_O_2_-induced production of ROS and upregulation of senescence-associated β-galactosidase activity. Using microRNA (miRNA) microarrays, the present study identified 24 miRNAs, which were differentially expressed in the troxerutin pretreated, H_2_O_2_-treated HDP cells. Subsequent prediction using bioinformatics analysis revealed that the altered miRNAs were functionally involved in several cell signaling pathways, including the mitogen-activated protein kinase and WNT pathways. Overall, these results indicated that ROS-mediated cellular damage was inhibited by troxerutin and suggested that the use of troxerutin may be an effective approach in the treatment of alopecia.

## Introduction

Trihydroxyethylrutoside (troxerutin) is one of the flavonoid rutoside derivatives. It exhibits non-mutagenic properties and has a functional role in the treatment of chronic venous insufficiency (CVI) (1,2). A number of studies have demonstrated other beneficial effects of troxerutin, *in vitro* and *in vivo*, and it may be effective in reducing different cytotoxicities. In particular, troxerutin has been observed to exhibit an inhibitory effect on the neurotoxicity induced by high cholesterol mediated cognitive deficits, kainic acid-triggered excitotoxic damage and β-amyloid oligomerization (3–5). In addition, troxerutin has a photoprotective effect against ultraviolet B (UVB) radiation in human skin cells, including dermal fibroblasts and keratinocytes (6,7). Troxerutin also exerts a protective effect against γ-radiation in mice (8,9). Although the precise cellular mechanisms underlying the effects of troxerutin remain to be fully elucidated, these reports can be summarized as a single meaningful finding, that troxerutin inhibits the production of reactive oxygen species (ROS). *In vivo* investigations have demonstrated that CVI-bearing patients have increased levels of ROS, and troxerutin has a protective effect against oxygen-derived free radical scavengers on the endothelium in these patients (10,11). In addition, the aforementioned neurotoxicities are inhibited following troxerutin application by reducing the production of ROS (3,4,12). UVB and γ-radiation are known ROS stimulators (13,14), and a previous study demonstrated that troxerutin protects against radiation-induced lipid peroxidation (9). These studies suggest that this toxerutin may offer a novel therapeutic strategy for ROS-induced diseases.

Dermal papilla (DP) cells are located at the base of hair follicles and are important in the induction of growth and maintenance of epithelial cells, which are the predominant components of hair follicles (15). In response to hormonal changes, DP cells direct the follicular epithelial cells to enter the hair growth cycle, which involves anagen, an active growing phase; catagen, a short transitionary regressive phase; and telogen, a dormant resting phase (15). An increasing body of evidence has demonstrated excessive loss of viability and death of DP cells in balding regions of the scalp, compared with non balding regions, due to increased levels of 5α-reductase (16), a converting enzyme for androgenic hormones and intracellular ROS (17). In addition, previous reports have indicated that oxidative stress is generated by the exposure of androgen sensitive prostate cancer cells to high levels of androgens (18), and that lipid peroxides increase the levels of ROS and apoptosis of the hair follicle cells (19). Furthermore, DP cells in the balding scalp grow more slowly *in vitro*, compared with cells from the non balding scalp. The reduced proliferative activity of balding DP cells is associated with changes in the expression levels of senescence-associated (SA) β-galactosidase, oxidative stress markers, superoxide dismutase and catalase (20). These findings indicate that oxidative stress is important in the loss of DP cells and in hair production.

In the present study, the hypothesis that troxerutin inhibits ROS-mediated cellular dysfunction in human DP (HDP) cells was investigated. In addition, using micro (mi)RNA microarrays and bioinformatics analysis, the role of troxerutin in the regulation of the expression and mechanisms of specific miRNAs was evaluated. The present study aimed to examine troxerutin as a potential novel chemical agent for the preven tion and/or treatment of alopecia.

## Materials and methods

### Cell culture and viability

The HDP cells were purchased from Innoprot (Biscay, Spain) and cultured in Dulbecco’s modified Eagle’s medium, containing 10% fetal bovine serum (FBS; Thermo Fisher Scientific, Waltham, MA, USA) and 1% penicillin streptomycin (Gibco Life Technologies, Grand Island, NY, USA) at 37°C and 5% CO_2_. The cells were plated at a density of 4×10^3^/well in a 96-well plate. At 70–80% confluence, the cells were treated with troxerutin (Sigma-Aldrich, St. Louis, MO, USA) at concentrations ranging between 0 and 60 *μ*M for 24 h at 37°C. Subsequently, 10 *μ*l water soluble tetrazolium salt assay solution (EZ-Cytox Cell Viability Assay kit; Itsbio, Seoul, Korea) was added to each well and, following incubation for 30 min at 37°C, the optical density was measured at 490 nm using an iMark microplate reader (Bio Rad Laboratories, Inc., Hercules, CA, USA). To examine troxerutin mediated ROS protection, the cells were pretreated with troxerutin at the following concentrations: 0, 5, 10 and 15 *μ*M for 8 h. Subsequently, 750 *μ*M H_2_O_2_ was added to each well. Following incubation for 24 h at 37°C, cell viability was evaluated using an EZ-Cytox Cell Viability Assay kit. The level of cell viability (%) was normalized to that of 0.1% dimethyl-sulfoxide (DMSO; Sigma-Aldrich)-treated cells. Each experiment was repeated at least three times. The P-value was determined using Student’s t-test and P<0.05 was considered to indicate a statistically significant difference.

### Analysis of cell cycle

The HDP cells (2×10^6^), which had been treated with troxerutin and/or H_2_O_2_ were trypsinized using 0.25% trypsin-EDTA (Gibco Life Technologies), washed once with phosphate-buffered saline (PBS), and used for analysis. The cell cycle distribution was measured using propidium iodide (PI; Sigma-Aldrich) staining solution, containing 50 *μ*g/ml PI, 0.5% Triton X-100 (Sigma-Aldrich) and 100 *μ*g/ml RNase (Qiagen, Hilden, Germany). The cells were fixed in 70% cold ethanol (Merck Millipore, Darmstadt, Germany) and incubated for 1 h at −20°C. Subsequently, PI staining solution was added to the fixed cells, followed by incubation for 1 h in the dark at 37°C. The PI fluorescence intensity was detected using a BD FACSCalibur flow cytometer (BD Biosciences, San Jose, CA, USA). The mean PI fluorescence intensity was calculated based on the measurements of 10,000 cells using the FL2-H channel.

### Analysis of intracellular levels of ROS

The HDP cells (2×10^6^), which had been treated with troxerutin and/or H_2_O_2_ were washed with PBS and trypsinized. Intracellular ROS levels were measured using 2′7′-dichlorofluorescein diacetate fluorescent dye (DCF-DA; Sigma-Aldrich), as described previously (21). The cells were resuspended in 10 *μ*M DCF-DA and further incubated at room temperature for 1 h in the dark. The intensity of the resulting fluorescence was measured using a BD FACSCalibur flow cytometer (BD Biosciences). The mean DCF fluorescence intensity was calculated based on measurements of 10,000 cells using the FL1-H channel. The M1 range was calculated as the percentage of each subpopulation of cells exhibiting increased DCF-DA fluorescence.

### Analysis of cellular senescence

The HDP cells (2×10^6^), which had been treated with troxerutin and/or H_2_O_2_ were washed in PBS and fixed for 5 min at room temperature in 2% formaldehyde/0.2% glutaraldehyde (Sigma-Aldrich). The cells were washed with PBS and incubated for 15 h at 37°C with SA β-galactosidase staining solution (BioVision, Milpitas, CA, USA). The stained cells (blue) were observed using a bright-field microscope (CKX41; Olympus Corporation, Tokyo, Japan; magnification, ×200), counted in three different fields and the percentage of stained cells was determined.

### Analysis of miRNA expression profiles

The HDP, which had been cells treated with troxerutin and/or H_2_O_2_ were washed in cold PBS and trypsinized for RNA purification. The total RNA was extracted and purified from the cells using TRIzol^®^ reagent (Invitrogen Life Technologies, Carlsbad, CA, USA), according to the manufacturer’s instructions. The integrity (RNA integrity number >8.0) and purity (A260/280 and A260/230 values >1.8) were confirmed using an Agilent 2100 Bioanalyzer^®^ (Agilent Technologies, Inc., Santa Clara, CA, USA) and a MaestroNano^®^ microvolume spectrophotometer (Maestrogen, Las Vegas, NV, USA), respectively. Samples (100 ng) of RNA meeting these criteria were first dephosphorylated by incubation with calf intestinal alkaline phosphatase (Agilent Technologies, Inc.) at 37°C for 30 min. Subsequently, cyanine 3-pCp labeling solution (Agilent Technologies, Inc.) and T4 RNA ligase (Agilent Technologies, Inc.) were added to the dephosphorylated RNA samples and incubated at 16°C for 2 h. Following the labeling reaction, the samples were dried and treated with GE Blocking Agent (Agilent Technologies, Inc.). The samples were hybridized to the SurePrint G3 Human v16 miRNA 8×60 K (Agilent Technologies, Inc.) microarray at 55°C, with constant rotation at 20 rpm in an Agilent Microarray Hybridization Chamber(Agilent Technologies, Inc.) for 20 h. The array was then washed and scanned using an Agilent SureScan Microarray scanner and the images captured were quantified using Agilent Feature Extraction software (version 10.7; Agilent Technologies, Inc.). The data were analyzed with the assistance of GeneSpring GX software version 7.3 (Agilent Technologies). In addition, fold-change analysis was performed to select those with ≥2.0-fold between the H_2_O_2_-treated control cells and those treated with troxerutin and H_2_O_2_.

### Bioinformatic analysis of altered miRNAs

Analysis of the biological significance of the altered miRNAs in the present study was performed, as previously described (21). First, the putative target genes of the altered miRNAs were predicted using MicroCosm Targets Version 5 (http://www.ebi.ac.uk/enright-srv/microcosm/htdocs/targets/v5/). Subsequently, the target genes were grouped into four categories: Aging, skin development, apoptosis and cell proliferation, based on the AmiGo 2 Gene Ontology (GO) analysis tool (amigo.geneontology.org/cgi-bin/amigo/browse.cgi). The putative target genes of each miRNA were further analyzed for biologic function using the Kyoto Encyclopedia of Genes and Genomes (KEGG) pathway within the Database for Annotation, Visualization and Integrated Discovery, (DAVID; http://david.abcc.ncifcrf.gov/home.jsp) bioinformatics resources (version 6.7), according to the standard procedures (22). The ‘KEGG_pathway’ category was processed by setting the threshold of the EASE score, a modified Fisher’s exact P-value, to 0.1. The KEGG pathways, identified as having a percentage of involved target genes / total target genes in each pathway >1% were selected.

## Results

### H_2_O_2_-induced cell damage is inhibited by troxerutin in HDP cells

**Figure 1:**
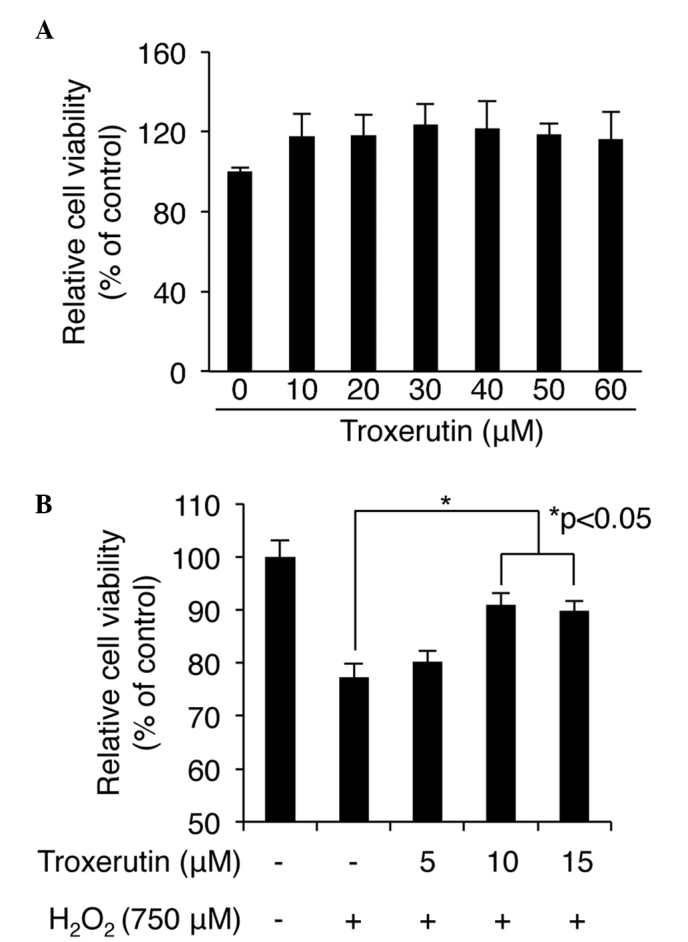
Troxerutin protects HDP cells against the H_2_O_2_-mediated reduction in viability. The viability of the (A) HDP cells treated with the indicated doses of troxerutin and in (B) HDP cells pretreated with the indicated doses of troxerutin followed by treatment with H_2_O_2_, were assessed. The results are presented as the mean ± standard derivation of three independent experiments. ^*^P<0.05, compared with control H_2_O_2_-treated sample. HDP, human dermal papilla.

HDP cells are a major component of skin and direct hair growth, and loss or senescence of these cells is a key cause of hair loss (15). Previous reports have demonstrated that cisplatin-and androgen overexposure-mediated cellular dysfunction, including apoptosis, can induce alopecia and occur predominantly by stimulating the production ROS in HDP cells (16,23,24). In our previous study, pretreatment of human dermal fibroblasts and HaCaT keratinocytes with troxerutin was observed to protect against UVB-mediated cell death (6,7). UVB light is an important inducer of ROS production in several types of cell (13); therefore, the present study examined the possible role of troxerutin in protecting against ROS-induced cell stress and damage in HDP cells. Initially, troxerutin-mediated cytotoxicity in HDP cells was screened for. No significant changes in cell viability were detected following treatment with between 0 and 60 *μ*M troxerutin for 24 h (Fig. 1A), indicating that troxerutin was non toxic in the HDP cells. Subsequently, the protective role of trox erutin against ROS-induced cell damage was determined using H_2_O_2_, a ROS inducer. The HDP cells were pretreated with several concentrations (0–60 *μ*M) of troxerutin for 6 h, followed by the addition of 750 *μ*M H_2_O_2_ and incubation for an additional 24 h. The results revealed that the maximum protective effect against ROS induced cell damage in the HDP cells occured folowing pretreatment with 10 *μ*M troxerutin (Fig. 1B). Treatment with H_2_O_2_ alone decreased cell viability to 77.33±2.44%; however, pretreatment with 10 *μ*M troxerutin maintained cell viability at 90.88±2.24% following H_2_O_2_ exposure (P<0.05; Fig. 1B). Concentrations of troxerutin >15 *μ*M did not significantly enhance the protective effect of 10 *μ*M troxerutin (Fig. 1B and data not shown). These results suggested that pretreatment with troxerutin inducedresistance against H_2_O_2_-mediated cytotoxicity in the HDP cells.

### H_2_O_2_-induced cell death is inhibited by troxerutin in HDP cells

**Figure 2:**
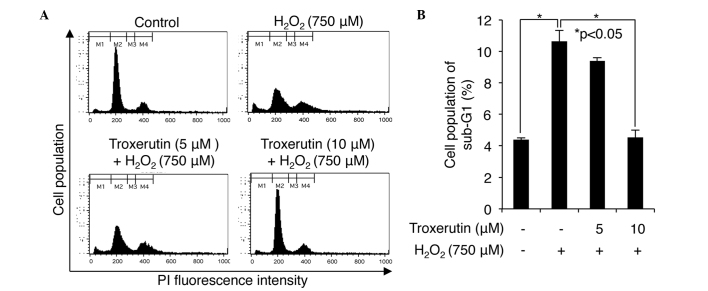
Troxerutin inhibits G2 phase arrest and cell death induced by H_2_O_2_ exposure in HDP cells. (A) Flow cytometric analysis was performed to determine the cell cycle distribution of the control HDP cells, HDP cells treated with H_2_O_2_ only and HDP cells pretreated with the indicated doses of troxerutin followed by treatment with H_2_O_2_. The Sub-G1, G1, S, and G2/M phases were separated using the indicated gates (M1, M2, M3 and M4). (B) Quantification of the percentages of the cell populations in the sub-G1 phase. The results are presented as the mean ± standard deviation of three independent experiments. ^*^P<0.05, compared with the control or H_2_O_2_-treated cells for indicated pairs. HDP, human dermal papilla; PI, propidium iodide.

Our previous study demonstrated that troxerutin effects the level of cell death induced by UVB irradiation (6,7) and Fig. 1 shows the protective role of troxerutin against ROS-induced cell damage, observed in the present study. Therefore, to examine whether troxerutin is involved in the response to ROS stress, which is known to inhibit cell-cycle progression and induce cell death, the present study investigated changes in the cell cycle and in cell death in the HDP cells pretreated with different concentrations of troxerutin prior to H_2_O_2_ exposure. The HDP cells were pretreated with 0, 5 or 10 *μ*M troxerutin for 6 h, followed by treat ment of the cells with 750 *μ*M H_2_O_2_ for an additional 24 h. Subsequently, the cells were analyzed by flow cytometry. At concentrations of 5 and 10 *μ*M, pretreatment with troxerutin caused a decrease in the number of cells in the sub G1 phase, indicative of cell death (Fig. 2A). H_2_O_2_ increased the percentage of the non-pretreated cells in the sub-G1 phase to 10.63±0.43%; however, this value increased to only 9.38±0.11 and 4.53±0.53% in the cells pretreated with 5 and 10 *μ*M troxerutin, respectively (P<0.05; Fig. 2A and B). Therefore, these results suggested that troxerutin overcame the effect of H_2_O_2_-mediated cell death, resulting in a diminution of cells in the sub-G1 phase.

### Hydrogen peroxide-induced ROS production is inhibited by troxerutin in HDP cells

**Figure 3:**
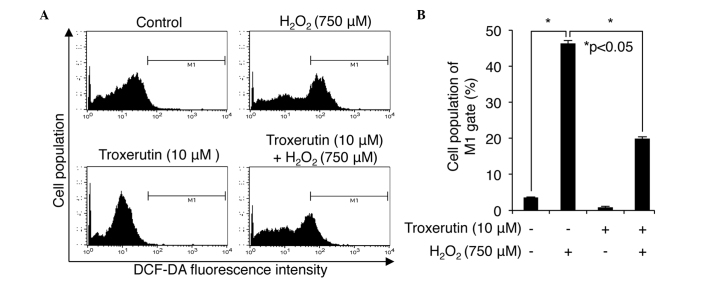
Troxerutin downregulates the level of ROS in HDP cells. (A) Flow cytometric analysis of the intracellular ROS levels in the control HDP cells, HDP cells treated with H_2_O_2_ only and the HDP cells pretreated with the indicated doses of troxerutin followed by treatment with H_2_O_2_. M1 indicates the subpopulation of cells emitting DCF-DA fluorescence signal. (B) Quantification of the percentage of cells in M1. The results are presented as the mean ± standard deviation of three independent experiments. ^*^P<0.05, compared with the control or H_2_O_2_-treated sample for indicated pairs. HDP, human dermal papilla; ROS, reactive oxygen species; DMSO, dimethyl sulfoxide; DCF-DA, 2′7′-dichlorofluorescein diacetate.

Although the above results indicated that troxerutin had a protective effect against H_2_O_2_-mediated cell death, it remained to be elucidated whether troxerutin also regulates the level of intracellular H_2_O_2_-induced ROS in HDP cells. To examine this, the presents study performed a fluorescent DCF-DA staining assay following treatment with troxerutin and H_2_O_2_ in the HDP cells. The DCF-positive cells were then analyzed using flow cytometry to determine and compare the levels of intracellular ROS in the control cells, non-troxerutin pretreated cells, troxeruti only treated cells and troxerutin pretreated/H_2_O_2_ treated cells. As shown in Fig. 3, in the control and troxerutin-only-treated cells, 3.58±0.15 and 0.89±0.11% were DCF-positive (P<0.05; Fig. 3B), suggestive of ROS respectively, whereas treatment with H_2_O_2_ alone increased the level of ROS to 46.36±2.33%. The cells pretreated with troxerutin were 19.92±1.95% DCF-positive following H_2_O_2_ treatment, indicating that troxerutin reduced the H_2_O_2_-induced production of ROS in the HDP cells.

### Hydrogen peroxide-induced senescence is inhibited by troxerutin in HDP cells

**Figure 4:**
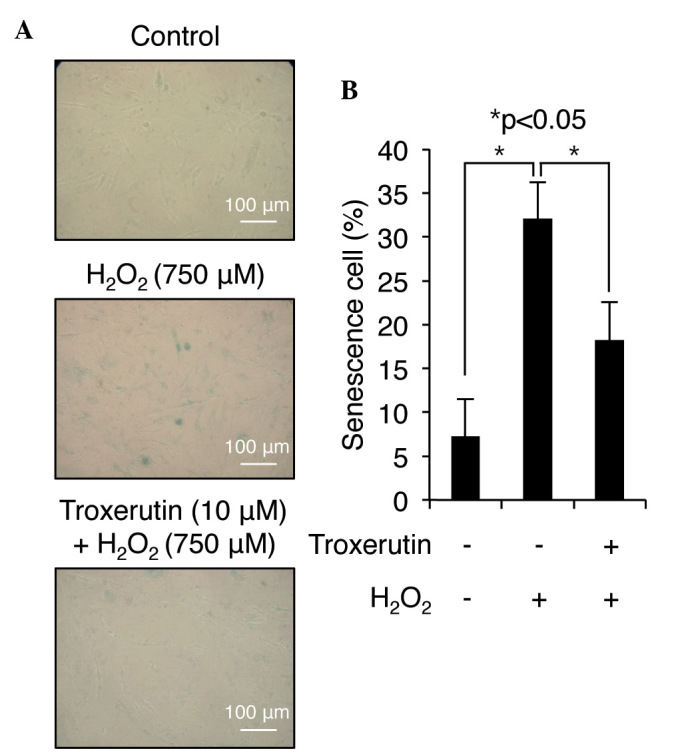
Troxerutin decreases H_2_O_2_-induced senescence in HDP cells. (A) Representative micrographs of control dimethyl sulfoxide-treated HDP cells, HDP cells treated with H_2_O_2_ only and HDP cells pretreated with the indicated dose of troxerutin followed by treatment with H_2_O_2_. The cells were stained for the presence of senescence-associated SA β-galactosidase activity. (B) Quantification of the percentage of senescent (SA β-galactosidase positive) cells. The results are presented as the mean ± standard deviation of three independent experiments. ^*^P<0.05, compared with the control or H_2_O_2_-treated cells for indicated pairs. HDP, human dermal papilla.

Increased ROS are one of the key mediators of cellular senescence (25). A previous report demonstrated that the premature senescence of balding DP cells is associated with changes in the expression of SA β-galactosidase and also suggested that oxidative stress may be involved in the premature senescence of these cells (20). The present study assayed for the presence of SA β-galactosidase activity to investigate whether troxerutin affects H_2_O_2_-induced senescence, thereby contributing to its protective effect against ROS-mediated cell damage. H_2_O_2_ treatment increased the number of SA β-galactosidase-positive cells to 32.11±3.32% compared with the control; however, only 18.22±5.21% of the cells pretreated with troxerutin were SA β-galactosidase-positive following treatment with H_2_O_2_ (Fig. 4). These data indicated that troxerutin has the potential to inhibit cellular senescence in HDP cells.

### Troxerutin-mediated protective effects against ROS are involved in changes in miRNA expression in HDP cells

The present study also analyzed the miRNA expression profiles of non-pretreated and troxerutin-pretreated HDP cells treated with H_2_O_2_, as miRNAs can be involved in cell death, ROS scavenging and senescence (21,26–28). In the microarray, a total of 24 miRNAs were detected with a ≥2.0-fold change in expression levels between the two groups. Among these, 10 miRNAs were upregulated and 14 were downregulated in the H_2_O_2_-treated cell, which had been pretreated with troxerutin (Table I). To investigate the biological value of the microarray data, several bioinformatic analyses were performed to predict the putative target genes of the altered miRNAs, and the GO and signaling pathways of the target genes. The putative target genes of each miRNA were deterfmined using MicroCosm Targets Version 5, following which GO analysis was performed for the target genes. Subsequently, the target genes of each miRNA were categorized into four biological functions: Aging, skin development, apoptosis and cell proliferation. Several target genes of each miRNA were found to be involved in these four biological functions at different levels (Tables II and III). For example, has-miR-602, which was the most highly upregulated miRNA (6.91-fold) based on the microarray data, potentially targets 34 genes, six of which were involved in aging, and one of the remaining 28 target genes was involved in skin development (Table II). Similarly, the target genes of the downregulated miRNAs were also differentially involved in the four functions (Table III), indicating that the altered miRNAs identified by the microarray analysis had distinct biological roles associated with the protective effect of troxerutin in H_2_O_2_-treated HDP cells. Therefore, the present study further analyzed the signaling pathways associated with the upregulated and downregulated miRNAs using KEGG pathway analysis and the DAVID bioinformatics tool (22), the results of which are presented in Tables IV and V, respectively. The results demonstrated that the altered miRNAs are functionally involved in shared and unique pathways among the miRNAs. For example, hsa-miR-602 was identified to be functionally involved in MAPK, insulin, and calcium signaling pathways, whereas has-miR-205 3p was found to be involved in cancer, MAPK, Wnt and cell adhesion signaling pathways. Overall, these results indicated that the miRNA expression patterns of non-pretreated and troxerutin-pretreated H_2_O_2_-treated HDP cells can be distinguished, and those which are significant changed may be involved in troxerutin-mediated protection against H_2_O_2_-induced cellular stress through the regulation of multiple signaling pathways.

## Discussion

In the present study, the protective effect of troxerutin against H_2_O_2_-induced oxidative stress in HDP cells was confirmed using biochemical assays. Notably, pretreatment with troxerutin decreased the cell death, ROS production and cellular senescence, which was mediated by exposure to H_2_O_2_. Although the specific signaling pathways involved in the protective effect were not demonstrated, the findings of the present study are important in that they identify troxerutin as a candidate agent for use in the prevention and/or treatment of alopecia. A growing body of evidence suggests the role of oxidative stress in alopecia, and that the prevention of oxidative stress may offer novel strategies for the intervention and reversal of alopecia and even graying of hair (17). A previous case study confirmed increased oxidative stress in alopecia areata patients compared with healthy individuals (29). In addition, a study using a mouse model demonstrated that hair dye-induced hair loss is caused predominantly by H_2_O_2_-induced oxidative stress (30). Oxidative stress stimulates the production of a known inhibitor of hair follicles, tumor growth factor-β, in DPC cells, which induces the onset of androgenic alopecia (24). Our previous studies demonstrated that troxerutin has a photoprotective effect against UV radiation on dermal fibroblasts and keratinocytes (6,7), and several clinical and theoretical reports have revealed that UV radiation has negative effects on hair growth through the induction of oxidative stress, acute telogen effluvium and follicular micro-inflammation in follicular stem cells (31–33). Therefore, countering oxidative stress can be considered an important strategy to overcome stress-or androgen-dependent alopecia, and the results of the present study confirmed that troxerutin inhibited oxidative stress-induced cellular damage in the DPC cells. In addition, our previous studies and the present studies demonstrated low levels of cytotoxicity of troxerutin on dermal fibroblasts, keratinocytes and DP cells (6,7). Therefore, further investigation of the clinical effect of topical application of troxerutin to the scalp is required.

Using miRNA microarray analysis, the present study identified 24 miRNAs in the HDP cells treated with troxerutin and H_2_O_2_, which were differentially expressed compared with the cells treated with H_2_O_2_ only. Of these, has-miR-602 was the most markedly upregulated by troxerutin in the H_2_O_2_-treated HDP cells (6.91-fold), and has been reported to downregulate the expression of the RASSF1A and TP73 tumor suppressor genes (34). Several reports have revealed that H_2_O_2_ induces the expression of TP73 (35) and that the anticancer drug cisplatin, which has been reported to induce alopecia in patients, stimulates ROS-induced apoptosis and functionally upregulates the expression of p73 (36–38). Although the cellular functions of miR-602, RASSF1A and TP73 have not been investigated in DP cells, the data of the present study suggested that the interaction between miR-602 and the two genes may be functionally involved in ROS-induced cellular stress and even alopecia-associated mechanisms. The biological functions of has-miR-575, which was the most downregulated miRNA in the results of the present study, have not been reported previously; however, it may regulate H_2_O_2_-mediated cellular stress. PDPK1, also termed PDK1, is a putative target of miR-575 (Table III) and a well-known kinase, which phosphorylates and activates Akt1 kinase, induces cell proliferation and protects against H_2_O_2_-induced apoptosis (39,40). The present study also classified the biological functions of differentially expressed miRNAs by troxerutin in the H_2_O_2_-treated HDP cells. KEGG pathway analysis of the target genes of the upregulated and downregulated miRNAs revealed 18 and 23 pathways, respectively, were statistically enriched. Among these, the WNT and MAPK signaling pathways, which were the most markedly enriched pathways associated with the target genes of the upregulated and downregulated miRNAs, are involved in the regulation of H_2_O_2_-mediated cellular stress, including apoptosis and antioxidative mechanisms (41–45), suggesting that the miRNAs altered by troxerutin may be involved in protective mechanisms against H_2_O_2_-induced damage through the regulation of these pathways.

In conclusion, the present study revealed a novel role of troxerutin as a putative antioxidant agent in HDP cells. In addition, the results revealed 24 differentially expressed miRNAs and determined the putative involvement of 18 signaling pathways associated with upregulated miRNAs and 23 signaling pathways associated with downregulated miRNAs in the troxerutin mediated protective effect against H_2_O_2_-induced cell damage. Although further experiments are required to confirm the differentially expressed miRNAs and their target genes, the results of the present study may assist in elucidating the mechanism underlying the troxerutin-mediated protection and miRNA-associated signaling pathways in HDP cells.

## Figures and Tables

**Table I:** MicroRNAs with ≥2-fold change in expression in troxerutin pretreated H_2_O_2_-treated human dermal papilla cells.

microRNA	Change relative to control	Direction of regulation	Chromosome
hsa-miR-150-3p	4.13	Up	19
hsa-miR-181a-2-3p	2.31	Up	9
hsa-miR-205-3p	4.28	Up	1
hsa-miR-21-3p	2.92	Up	17
hsa-miR-29b-1-5p	3.72	Up	7
hsa-miR-3127-5p	2.19	Up	2
hsa-miR-371a-5p	2.30	Up	19
hsa-miR-3663-3p	2.53	Up	10
hsa-miR-4298	2.01	Up	11
hsa-miR-602	6.91	Up	9
hsa-miR-1181	−3.14	Down	19
hsa-miR-1202	2.78	Down	6
hsa-miR-1224-5p	−4.66	Down	3
hsa-miR-1290	2.15	Down	1
hsa-miR-135a-3p	5.61	Down	3
hsa-miR-28-5p	2.95	Down	3
hsa-miR-378a-5p	2.01	Down	5
hsa-miR-4271	−2.16	Down	3
hsa-miR-452-5p	−2.51	Down	X
hsa-miR-572	−4.26	Down	4
hsa-miR-575	−8.01	Down	4
hsa-miR-629-3p	3.12	Down	15
hsa-miR-939	2.22	Down	8
hsa-miR-940	−2.30	Down	16

miR, microRNA.

**Table II:** Predicted targets of microRNAs upregulated in response to troxerutin pretreatment in H_2_O_2_-exposed human dermal papilla cells.

microRNA	Aging	Skin development	Apoptosis	Cell proliferation
hsa-miR-150-3p	–	–	BCL3, INHBA, ARHGEF2, RHOA, ATG7, MAP3K5, MECOM, PLAGL1	BCL3, INHBA, MECOM, ARHGEF2, RHOA, NDN, PROX1, BTRC, TGFBI
hsa-miR-181a-2-3p	LMNA, SRF	SRF	LMNA, MED1, BDNF, TIAL1, SRPK2, CITED2, AGAP2, PSMD7, MAPK8	SRF, MED1, BDNF, TIAL1, SRPK2, CITED2, SOX11, FBXW7
hsa-miR-205-3p	TBX3, CDK6, MNT, IL1B, TECP2L1, PNPT1, ATR	APC, DBI	BRCA1, HDAC2, SOS2, SIX4, GRAM, MDM4, CUL5, NBN, MNT, IL1B, TBX3, WNT5A, RAD21, MAP3K5, RASSF6, CREB1, GLO1, API5, SOS1, APC, MSX2, FGF2, SOX2, DUSP1, GSK3B, PSMA5,MITF, HiPK2, HOXA13, PARK7, NAIP, BCLAF1	MITF, GRAM, MDM4, CUL5, NBN, CDK13, CASK, PURA, MNT, IL1B, TBX3, HDAC2, CDK6, MSX2, FGF2, SOX2, HiPK2, BRCA1, WNT5A, EVI5, TOB1, NUMB
hsa-miR-21-3p	CDK6	–	MAP2K4, MAP3K1, BCL2L11, SMAD3, CUL3, SOX4, BAG4, RNF41, AMIGO2, SLC11A2, KDM28, DAB2IP, FOXO3, CCAR1, ROBO2, TRIM32, DSG1	CUL3, SOX4, NR6A1, FTO, TRIM32, FOXO3, SMAD3, CDK6, KDM28, DAB2IP, CD274, PBRM1
hsa-miR-29b-1-5p	NR3C1, SIRT1	–	NR3C1, SIRT1, REST, PTK2, SOS2, NUAK2, PSMD7	NR3C1, SIRT1, REST, PTK2, FGF18, INSR, PBRM1
hsa-miR-3127	–	–	–	–
hsa-miR-371a-5p	–	LEF1, ATP7A, COL8A1	LEF1, SOX2, CITED2, STK4, RB1CC1, BARD1, GSK3B, PSMF1, NR4A2, DYRK2, RPS6KA1, ITSN1, MAP3K1	LEF1, SOX2, CITED2, STK4, COL8A1, RNF10, MAPRE1, BTG3, CCR2, FRS2, PRMT5
hsa-miR-3663-3p	FAS, CASP2, CDKN1A, PTH1R	ADAMTS2, BCL11B, COL3A1, COL1A1	FAS, CASP2, BCL11B, USP28, TGFB2, DDX5, COMP, PIGT, CDKN1A, TIAL1, PPP2R1B, PSMA2, MEF2D	FAS, TIAL1, TGFB2, USP28, CDKN1A, BCL11B, VSIG
hsa-miR-4298	HMGA1, AMFR	–	MED1, FGF2, TRAF5, CCAR1	HMGA1, MED1, WT1, FGF2
hsa-miR-602	EDN1, VDR, SOD2, HTT, SLC34A2, CHEK1	APC	NOG, ERBB4, PSMD2, PIM1, EDN1, VDR, SOD2, DYRK2, ALDH1A2, CLI2, SEMA3A, HTT, APC, H1F0, PPARG, BCL2L15, JMY, TP53BP2, MYO18A, SHF	NOG, ERBB4, PIM1, PPARG, CLI2, CDC27, CDK13, LIFR, EDN1, VDR, SOD2, STAT3, APC, ALDH1A2, ACSL6, PPP1R8, EMX2, CDK9, RTKN2, ID4, ZEB1

miR, microRNA.

**Table III:** Predicted targets of microRNAs downregulated in response to troxerutin pretreatment in H_2_O_2_-exposed human dermal papilla cells.

microRNA	Aging	Skin development	Apoptosis	Cell proliferation
hsa-miR-1181	–	–	–	–
hsa-miR-1202	CLNB, PNPT1, SLC18A2	–	CLN8, RRN3, PIK3CG, ETS1, DRAM1, DNAJC10, STEAP3, IKBKG, SOS1, NOD1	RRN3, PIK3CG, ETS1, CDC6, BCAT1, NRP1, ERG, SESN1, FZD6, CD276, GAS8, RPS15A
hsa-miR-1224-5p	HMGA2, AQP2, SLC1A2	APC	HMGA2, AQP2, APC, FGFR1, ADORA1, SATB1, STAT5B	RBBP7, APC, CD160, RC3H1, HMGA2, FGFR1, ADORA1, SATB1, STAT5B, NOLC1
hsa-miR-1290	HMGA2, NUAK1, TERF2, SLC1A2, FADS1, DDC	APC, COL8A1	HMGA2, APC, RRN3, ITGAV, CSE1L, NOTCH1, GAS, BMI1, FOXC1, ROBO1, USP28	HMGA2, BMI1, NUAK1, APC, MLL2, RRN3, ITGAV, CSE1L, NOTCH1, GAS, HES1, NPR3, CDC27, COL8A1, CDKN2B, FOXC1, ROBO1, USP28, FIGF, NRAS
hsa-miR-135a-3p	–	TFAP2A	TFAP2A, POU3F3, RRP8, PEG3, DYRK2,	TFAP2A, POU3F3, DERL2, RERG, COL8A1, CEP120
hsa-miR-28-5p	–	–	MST4 CNTFR, STK4, BAG1, SON, NR4A3, PAK2	CNTFR, STK4, HTR4, FTSJ2, SESN1, TNS3, RAP18, DERL2
hsa-miR-378a-5p	PML	–	DFFA, ITSN1, CTSB, ROBO2, DEPTOR, RAG1, RFFL, IL24, PML, VHL, FRZB, STK4, BAG1, ITGB2	FZD3, RAC2, CCND2, FER, PML, VHL, FRZB, STK4, NUDC, PDAP1, ITGAL, PELI1, HNF4A, CD33
hsa-miR-4271	HMGA1, AMFR, SLC6A3	–	ALDH1A2, SPN, EIF2AK3, FOXO3, WNT7B, MAPK1, CYLD, MAPT, MEF2D, DAPL1, EP300	COL4A3BP, FOXO4, PDGFB, WNT7B, MAPK1, ALDH1A2, CDK2, SPN, MXD1, FOXO3, TGFBR3, CNOT8, MBD2, CD209, CDON, HOXD13,
hsa-miR-452-5p	TIMP3	–	SPRY2, PAX3, SOX7, LRP6, SNAI2, CSNK2A2, FGD4, PKN2, ITGA6, PDCD6IP	SPRY2, PAX3, SOX7, LRP6, SNAI2, RPA1, EPS8, NFIB, MAPRE1, ODZ1, CDCA7L, CD47, E2F3, PURA,
hsa-miR-572	–	–	HIP1, CASP10, E2F2, MAP3K1	RUNX1, CDC27, ROS1
hsa-miR-575	–	–	ZBTB16, HIP1, PDPK1, BRAF, CASP10, E2F2, MAP3K1, DNM1L	ZBTB16, NR3C2, NDEL1, ROS1, BRFOX2, KIF15
hsa-miR-629-3p	SOD2, VDR, EDN1, CHEK1, SLC34A2	–	THOC1, MYO18A, TP53BP2, APC, PPARG, PIM1, PSMD2, SOD2, VDR, EDN1, ERBB4, PERP, BCL2L15	DLG3, RTKN2, CDK9, STAT3, EPHB1, ACSL6, LIFR, EREG, APC, PPARG, PIM1, STAT6, PDGFC, ZEB1, NOLC1, ID4, SOD2, VDR, EDN1, ERBB4, CDK13, CDC27
hsa-miR-939	TIMP1, ATM, CDKN1A, NEK6, SCL34A2, PRELP, SLC1A2	NGFR, COL1A1	TNF, BCL6, BTC, NRG1, IHH, TIMP1, ATM, WNK3, CLIP3, NEK6, NGFR, MT3, TRAIP, CDKN1A, NACC1, IP6K2, PAX7, CAMK1D, CASP10, USP7, CSNK2A2, THRA, INHBB, BCL2L2	BCL6, BTC, NRG1, IHH, GRN, TRAIP, CDKN1A, TNF, E2F8, RXRB, RARA, DRD2, CSF1, TIMP1, ATM, NGFR, MT3, NOS2, AGGF1, ELN
hsa-miR-940	–	–	–	–

miR, microRNA.

**Table IV:** Functional annotation chart for miRNAs upregulated in response to troxerutin pretreatment in H_2_O_2_-exposed human dermal papilla cells.

microRNA	Putative target genes (n)	KEGG pathway	Genes involved in the term (n)	Involved genes/total genes (%)	P-value
miR-150-3p	184	Wnt signaling pathway	5	2.7	6.00E-02
		Neurotrophin signaling pathway	4	2.2	1.20E-01
		Ubiquitin mediated proteolysis	4	2.2	1.50E-01
		Adherens junction	3	1.6	1.70E-01
miR-181a-2-3p	189	Endocytosis	6	3.2	2.90E-02
		Chemokine signaling pathway	6	3.2	3.00E-02
		Ubiquitin mediated proteolysis	5	2.6	3.90E-02
		Pancreatic cancer	4	2.1	3.00E-02
		Adherens junction	4	2.1	3.50E-02
		Nucleotide excision repair	3	1.6	6.40E-02
miR-205-3p	944	Pathways in cancer	19	2.0	2.50E-01
		MAPK signaling pathway	17	1.8	1.70E-01
		Wnt signaling pathway	15	1.6	9.20E-03
miR-21-3p	210	Cell adhesion molecules	7	3.3	4.70E-03
		Ubiquitin mediated proteolysis	6	2.9	2.30E-02
		Long-term potentiation	5	2.4	8.60E-03
		Oocyte meiosis	5	2.4	4.20E-02
miR-29b-1-5p	265	Insulin signaling pathway	5	1.9	8.50E-02
		Cell cycle	4	1.5	2.00E-01
		Wnt signaling pathway	4	1.5	2.90E-01
		Jak-STAT signaling pathway	4	1.5	3.00E-01
mir-3127-5p	205	–	–	–	–
miR-371a-5p	351	Spliceosome	8	2.3	4.20E-03
		Wnt signaling pathway	7	2.0	3.60E-02
mir-3663-3p	305	MAPK signaling pathway	12	3.9	5.90E-03
		Pathways in cancer	11	3.6	5.50E-02
		Neurotrophin signaling pathway	7	2.3	2.00E-02
		Pancreatic cancer	5	1.6	3.50E-02
		Chronic myeloid leukemia	5	1.6	4.00E-02
mir-4298	185	Oocyte meiosis	5	2.7	8.70E-03
		Neuroactive ligand receptor interaction	5	2.7	1.20E-01
		Calcium signaling pathway	4	2.2	1.40E-01
		Phosphatidylinositol signaling system	3	1.6	1.10E-01
miR-602	302	MAPK signaling pathway	7	2.3	2.20E-01
		Insulin signaling pathway	6	2.0	5.30E-02
		Alzheimer’s disease	6	2.0	1.00E-01
		Calcium signaling pathway	6	2.0	1.30E-01

miR, microRNA; MAPK, mitogen activated protein kinase; JAK, Janus kinase; STAT, signal transducers and activators of transcription.

**Table V:** Functional annotation chart for miRNAs downregulated in response to troxerutin in H_2_O_2_-exposed HDP cells.

microRNA	Putative target genes (n)	KEGG pathway	Genes involved in the term (n)	Involved genes/total genes (%)	P-value
miR-1181	2	–	–	–	–
miR-1202	241	Pathways in cancer	8	3.3	1.50E-01
		Insulin signaling pathway	5	2.1	1.10E-01
		Phosphatidylinositol signaling system	4	1.7	7.60E-02
		ABC transporters	3	1.2	1.20E-01
		mTOR signaling pathway	3	1.2	1.50E-01
		Inositol phosphate metabolism	3	1.2	1.60E-01
miR-1224-5p	213	Axon guidance	4	1.9	1.00E-01
miR-1290	593	Pathways in cancer	17	2.9	4.00E-02
		Insulin signaling pathway	13	2.2	7.60E-04
		Regulation of actin cytoskeleton	12	2.0	6.30E-02
		MAPK signaling pathway	12	2.0	1.90E-01
		ErbB signaling pathway	11	1.9	2.80E-04
miR-135a-3p	140	–	–	–	–
miR-28-5p	157	MAPK signaling pathway	7	4.5	1.20E-02
		Axon guidance	4	2.5	6.60E-02
miR-378a-5p	366	Wnt signaling pathway	7	1.9	3.60E-02
		TGF-β signaling pathway	4	1.1	1.70E-01
miR-4271	361	Jak-STAT signaling pathway	7	1.9	7.80E-02
		Lysine degradation	4	1.1	5.20E-02
miR-452-5p	327	Oocyte meiosis	8	2.3	1.30E-03
		Wnt signaling pathway	7	2.0	2.60E-02
		ECM-receptor interaction	5	1.4	3.80E-02
		Small cell lung cancer	5	1.4	3.80E-02
miR-572	6	–	–	–	–
miR-575	241	MAPK signaling pathway	8	3.3	7.70E-02
		Prostate cancer	6	2.5	7.70E-03
		Melanoma	5	2.1	1.70E-02
		Cell cycle	5	2.1	9.60E-02
		Aldosterone-regulated sodium reabsorption	4	1.7	1.90E-02
		mTOR signaling pathway	4	1.7	3.50E-02
		Androgen and estrogen metabolism	3	1.2	9.40E-02
miR-629-3p	441	PPAR signaling pathway	6	1.4	1.20E-02
miR-939	365	Calcium signaling pathway	10	2.4	1.30E-02
		Regulation of actin cytoskeleton	9	2.1	8.90E-02
		ErbB signaling pathway	5	1.2	1.20E-01
		p53 signaling pathway	4	0.9	1.80E-01
		Wnt signaling pathway	6	1.4	2.20E-01
miR-940	–	–	–	–	–

miR, microRNA; mTOR, mammalian targets of rapamycin; MAPK, mitogen-activated protein kinase; ECM, extracellular matrix; Jak, Janus kinase; STAT, signal transducers and activators of transcription; TGF, transforming growth factor; PPAR, peroxisome proliferator-activated-receptor.
